# Human Milk Drives the Intimate Interplay Between Gut Immunity and Adipose Tissue for Healthy Growth

**DOI:** 10.3389/fimmu.2021.645415

**Published:** 2021-04-12

**Authors:** Lieke W. J. van den Elsen, Valerie Verhasselt

**Affiliations:** School of Molecular Sciences, The University of Western Australia, Perth, WA, Australia

**Keywords:** human milk, adipose tissue, growth, gut immunity, metabolic homeostasis

## Abstract

As the physiological food for the developing child, human milk is expected to be the diet that is best adapted for infant growth needs. There is also accumulating evidence that breastfeeding influences long-term metabolic outcomes. This review covers the potential mechanisms by which human milk could regulate healthy growth. We focus on how human milk may act on adipose tissue development and its metabolic homeostasis. We also explore how specific human milk components may influence the interplay between the gut microbiota, gut mucosa immunity and adipose tissue. A deeper understanding of these interactions may lead to new preventative and therapeutic strategies for both undernutrition and other metabolic diseases and deserves further exploration.

## Introduction: Health Benefits of Human Milk

Human milk is the most efficient way to prevent child morbidity and mortality related to respiratory and gastro-intestinal infectious disease (1). It also significantly reduces the incidence of necrotizing enterocolitis (2), a serious intestinal disease in preterm newborns. The time window for breastfeeding coincides with the rapid development of the infant and its immune system and there is accumulating evidence human milk influences the child’s growth trajectory and long-term health outcomes such as overweight and diabetes in later life (1, 3–5).

In this review, we discuss the role of breastfeeding in healthy growth and adipose tissue development as this is a largely unexplored area to date. Especially the contribution of individual human milk components on metabolic health remains poorly understood. Identifying human milk components that can aid in healthy postnatal growth and development could provide new preventative and therapeutic strategies for malnutrition in early life and long-term metabolic health.

## Regulation of Growth by Human Milk: Potential Mechanisms

As breastfeeding is physiological for the newborn, human milk is expected to be the diet that is most adapted to the growth needs of the infant and is therefore considered as the normative standard for infant nutrition. Breastfed infants increase more in weight, length, and fat during the first months of life, followed by a slower growth velocity up to one year of age, when compared to formula-fed infants (5). Human milk composition is extremely dynamic, and varies within a feed, diurnally, over lactation and between mothers (6, 7). The link between the nutrients and other bioactives in human milk and growth occurs at various levels, from appetite regulation to direct sensing of nutrients by immune cells in the intestine. In the next paragraphs, we will review the multiple pathways that human milk potentially acts on to improve growth and adipose tissue development.

### Protection from Infections

Infectious diseases are a major cause of growth failure (8). Therefore, the major impact of breastfeeding in early life on infectious disease prevention (1) is expected to improve growth outcomes. Indirectly, the lower antibiotic exposure in breastfed infants also has potential for profound improvement in growth as antibiotics in early life have been linked to altered growth outcomes (9–11).

### Regulation of Appetite

Healthy growth promoted by breastfeeding has been linked to the establishment of infant appetite regulation and feeding pattern development (12). Two key hormones found in human milk control appetite: leptin decreases while ghrelin increases appetite and are thus thought to be involved in the regulation of milk intake, energy balance and adiposity. However, studies reporting on leptin and ghrelin in human milk and infant’s weight gain and fat mass have shown inconsistent results, as discussed elsewhere (4).

### A Tailored Source of Macronutrients

Breastfeeding is likely to provide the neonate with the nutrient and energy content best suited to the early stages of life. The macronutrient composition of human milk varies within mothers and across lactation but is remarkably conserved across populations (6). Lactose, the primary sugar of human milk, is the most abundant and least variable macronutrient in human milk (6). Breast milk protein content adapts to the growth requirements of the infant (13) decreasing over the first 4-6 weeks of life (6, 7). The energy content of milk correlates with its fat content, which is the most variable macronutrient in milk. Fat levels are especially high in the hind milk (last milk of a feed) and in afternoon and evening feedings (6, 7). Milk lipids, consisting for >98% of triglycerides, are packaged in milk fat globules for delivery to the infant’s gastrointestinal tract. Bile salt-stimulated lipase (BSSL) is one of the key enzymes for lipid digestion in the neonate and is present in human milk to help the neonate with fat absorption. Inactive BSSL in pasteurized human milk leads to fat malabsorption and reduced growth rates in preterm infants (14). BSSL-deficient mice pups also demonstrate epithelial disruption and growth deficiency (14). Human milk also contains bile acids (15), which may aid in digestion and absorption of fat in the neonate. Circulating bile acids also activate transmembrane G protein-coupled receptor 5 (TGR5) and increase the secretion of glucagon-like peptide-1 (GLP-1) associated with decreased body weight (16). Thus, providing bile acids *via* breast milk may affect growth and body weight of the infant.

### Somatotropic Axis

Postnatal growth is driven by the activity of the somatotropic axis (17, 18). Newborns produce Growth Hormone (GH) in the pituitary gland which stimulates the production of Insulin-like Growth Factor-1 (IGF-1) by the liver and peripheral tissues. GH receptor and IGF1 receptor signaling promote organ and systemic growth (19). The infant also receives exogenous GH through breast milk. GH concentrations are higher in colostrum than in mature milk (20), and levels in the serum peak around birth and then gradually decline (19). IGF and IGF-binding proteins are also abundantly present in milk, especially in colostrum (20) but the role of IGF-1 in growth trajectories and adiposity in early life remains poorly understood with conflicting data reported (21). Most studies failed to provide evidence for sufficient absorption of IGF-1 to alter systemic IGF concentrations (22), which increase from birth to weaning (19). As such, direct contribution of milk-derived IGF-1 to somatic growth in infants seems unlikely (22) but it may be important for gut epithelium development and thereby nutrient absorption and gut integrity-related immune homeostasis (see below). Human milk may also increase systemic IGF-1 indirectly *via* branched amino acids present in milk proteins such as casein (22).

### Healthy Adipose Tissue Development

In humans, adipose tissue appears early in the second trimester of the pregnancy (23). Both white and brown adipose tissues are key for energy homeostasis. White adipocytes store excess energy as triglycerides and release these when needed. Adipocytes also secrete various hormones such as leptin, adiponectin, and resistin and produce adipokines (adipose cytokines) such as TNF-α, IL-1β and IL-6 regulating insulin activity and glucose metabolism and contributing to tissue energy homeostasis (24). On the other hand, brown adipocytes have the ability to burn off lipids as heat (25). These adipocytes can also emerge within white adipose tissue (WAT) and this so-called beige adipose tissue is abundant in early life and undergoes a reduction with age (25, 26). To understand the influence of human milk on growth it thus appears fundamental to elucidate whether human milk acts on adipose tissue development and function. Currently the knowledge is very scarce in this area.

#### Adipogenesis

During a process called adipogenesis, progenitor cells develop into preadipocytes and subsequently mature, lipid-laden adipocytes (27). Adipocyte progenitor cells are committed to becoming mature adipocytes in fetal and early postnatal life (28, 29). Leptin and adiponectin can stimulate the differentiation from preadipocytes into adipocytes *in vitro* (30, 31). It should however be noted that the literature reports contrasting findings for breast milk hormones and adipokines in relation to adiposity *in vivo*, which is discussed in a recent systemic review by Mazzocchi et al. (4). Further studies are needed to clarify the roles of bioactive components in breast milk in relation to body composition. Other breast milk factors such as lactoferrin (32) and short-chain fatty acids (SCFA) (33, 34) have also been found to increase adipogenesis *in vitro*. SCFA are microbiota-derived metabolites that are most likely produced by the maternal gut microbiota and enter the milk *via* the circulation (35). In addition, milk oligosaccharides are fermented in the colon of the neonate generating SCFA (36, 37). SCFA can stimulate adipogenesis through G protein-coupled receptors (GPCR) signaling (33) and inhibition of histone deacetylase activity (34). Thus, as epigenetic regulators SCFA may have the potential to program adiposity. On the other hand, inflammatory cytokines such a TNF-α reportedly inhibit adipogenesis (38, 39), highlighting the potential importance of anti-inflammatory compounds in human milk. While there are no studies today on the direct influence of human milk on adipose tissue development, we can postulate that the nutrient content and anti/pro-inflammatory profile of human milk will influence adipocyte ontogeny. This deserves to be addressed in future research.

#### Adipose Tissue Hypertrophy and Proliferation

Excess nutrients are stored in white adipocytes as lipid droplets in response to insulin (40). Studies in mice have identified that once adipocytes increase in size in response to excess nutrient load during the early post-weaning period, a subsequent expansion of progenitors occurs due to increased systemic IGF-1 levels (41). If these cells encounter another dietary challenge in adulthood they increase in numbers, resulting in higher adipose tissue mass (41). Protein restriction in mice at weaning resulted in reduced adipocyte proliferation and growth restriction. These young mice increased their food intake in order to adapt, and this continued into adulthood (42). In culture, adiponectin has been shown to promote proliferation and increase lipid content of adipocytes (31). These studies show the importance of the diet for lipid accumulation and adipocyte proliferation, suggesting a significant role for the growth-adapted energy and nutrient content of human milk for adipose tissue expansion in early life.

#### Beiging

Neonates are born with beige adipose tissue-dominated fat depots. The energy content of human milk has the potential to influence adipose tissue beiging, as it has been shown that restricting calorie intake in young mice reduces the expression of brown adipose tissue (BAT) markers such as uncoupling protein 1 (UCP-1) in WAT at weaning (43). Another study has demonstrated that a high-fat diet in young mice that were exposed to a low-protein diet prenatally prevented the differentiation of precursors into beige adipocytes (44). Leptin and adiponectin promoted beiging of WAT in mice (45, 46) and lactoferrin upregulated UCP-1 expression in brown adipocytes in culture (47). The lipid composition of human milk is key for beige adipose tissue development. A recent study in mice demonstrated that breast milk-specific alkylglycerols sustain beige adipocytes through adipose tissue macrophages. Macrophages metabolize the alkylglycerols into platelet-activating factor (PAF), which leads to IL-6 secretion. Subsequently IL-6/STAT3 signaling in adipocytes triggers beige adipose tissue development. Therefore, breast milk intake delays the reduction of beige fat in early life, and reduces fat accumulation (48). Importantly, these lipids are absent in formula or the adult diet and the replacement of beige adipose tissue by WAT is accelerated in obese children (49). In addition, human milk SCFA levels are inversely associated with infant weight gain and adiposity and thus might protect from excess weight gain in early life (50). Although the exact mechanisms remain to be determined, milk-derived SCFA may contribute to the regulation of adiposity *via* the activation of BAT increasing energy expenditure (51, 52). These studies suggest that human milk energy content and lipid composition are key for healthy adipose tissue development and energy expenditure.

#### Adipose Tissue Immune Homeostasis

The adipose tissue does not only comprise adipocytes as its stromal vascular fraction contains heterogeneous cell populations such as mesenchymal progenitor/stem cells, preadipocytes, endothelial cells, pericytes and immune cells. Data from adult experimental models highlight that these cells also play an important role in controlling adipocyte energy metabolism. Regulatory T cells (Treg) support adipose tissue function by keeping local and systemic inflammation in check and promoting insulin sensitivity (53). Both visceral and subcutaneous WAT have low fractions of CD4+ FoxP3+ Treg cells at birth. In the visceral WAT Tregs accumulate over time and account for over half of T cells in lean adult mice (54). Besides Treg, type 2 immunity is required in the adipose tissue for optimal energy metabolism as it leads to the activation of eosinophils and alternately activated M2-type macrophages. Together these cells act to promote browning, which can reduce adiposity (55, 56) as described above.

Human milk has the potential to assist in adipose tissue homeostasis by preventing chronic systemic inflammation, which is linked to preserving the gut mucosal barrier (discussed in more detail below). Reducing circulating lipopolysaccharide (LPS) concentrations lowers pro-inflammatory gene expression in visceral WAT, dampening local WAT inflammation and improving insulin sensitivity (57). As lipids do not only serve as an energy substrate, but also have an important role in cell signaling, the quality of lipids in human milk is of interest for the development and inflammatory status of the neonate. Long chain n-3 polyunsaturated fatty acids (PUFA) are generally considered protective against inflammation (58) and have been shown to decrease the infiltration and cytokine production of inflammatory macrophages in adult adipose tissue (59). Although the key role of PUFA for development in early life (e.g. for neural development) is widely recognized, its importance for growth and metabolic health in infancy remains unclear. The interaction of SCFA with adipose tissue is relevant for the prevention of metabolic disease and its associated inflammation (60). SCFA have anti-inflammatory properties and reduce the expression of inflammatory cytokines and chemokines as well as leukocyte infiltration in adipose tissue (61). SCFA however also induce leptin secretion, which can induce a proinflammatory cytokine profile (30, 60, 62). Lactoferrin has been demonstrated to decrease inflammatory markers in adipocytes (32). The relevance for this in early life remains unknown and requires further investigation. However, the examples above illustrate that mediators present in human milk, or generated in the infant’s gut from milk components, can support adipose tissue homeostasis.

### Gut (immune) Function and Metabolic Homeostasis

**Figure 1:**
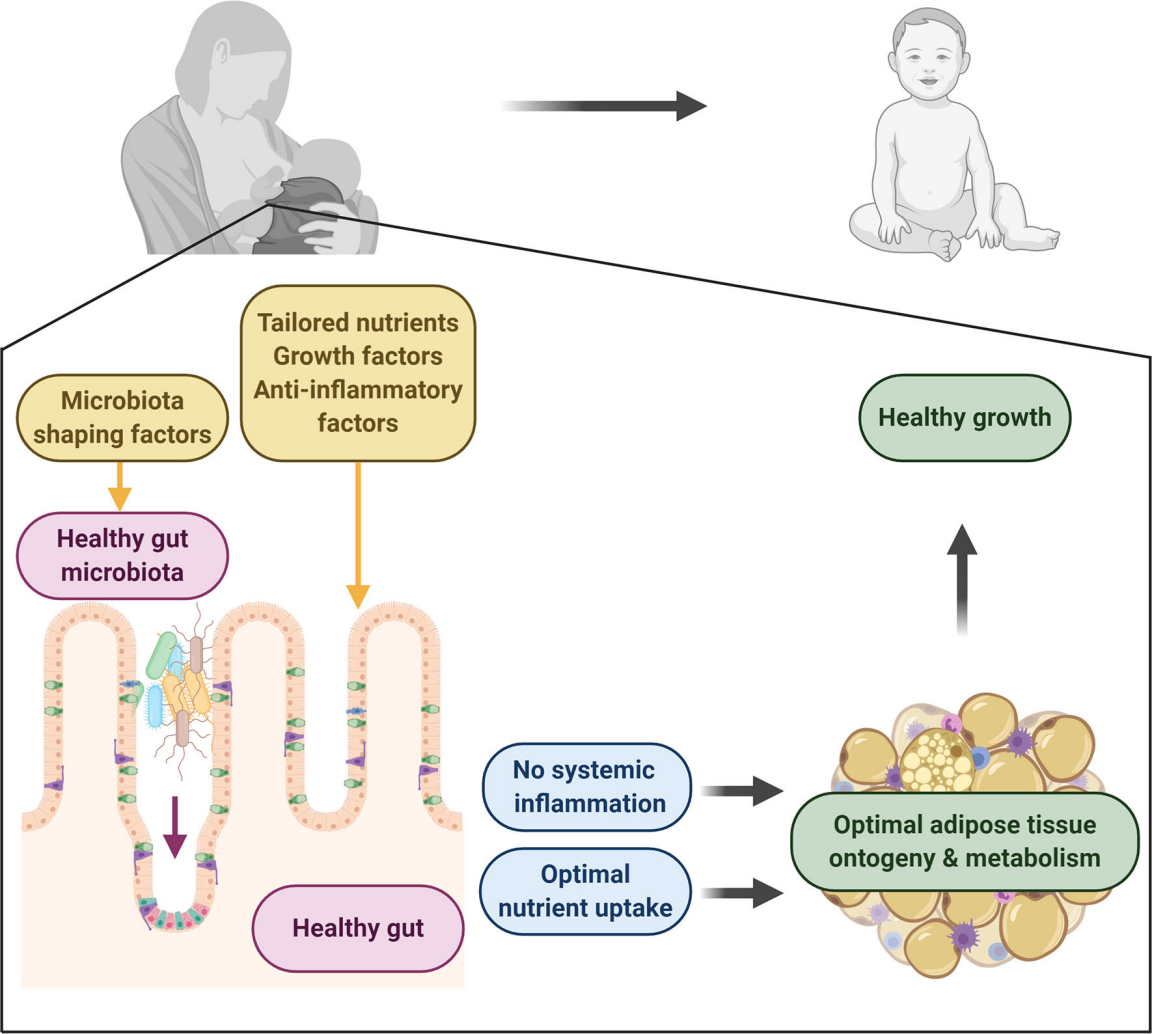
Human milk directs healthy adipose tissue development and growth. Human milk contains a plethora of factors with the potential to drive healthy development. It contains key macro- and micronutrients for the neonate to develop and thrive. It also provides important factors for the development of a healthy gut, which forms a selective immune barrier with the capacity to take up nutrients and control systemic inflammation. Microbiota-shaping factors in milk aid in the establishment of a healthy gut microbiota which contributes to healthy gut development and which regulatory metabolites can circulate, protecting from inflammation. Together this will lead to healthy adipose tissue development, leading to optimal growth in infants. Figure created with BioRender.com.

In the next paragraphs we will provide a more detailed overview about the link between human milk, gut (immune) function and metabolic health (see **Figure 1**). Research in adults, both in mice and humans, has demonstrated a key role for the gut in regulating metabolic homeostasis and energy storage. The intestinal mucosa is important for the uptake of food-derived nutrients and microbiota-derived metabolites. It also represents an immune barrier that prevents the invasion of pathogens and an inflammatory response to contents in the lumen. In addition, insulinotropic hormones (incretins) including GLP-1 and glucose-dependent insulinotropic polypeptide (GIP) are secreted from enteroendocrine cells and control glucidic metabolism. However, there is a lack of literature on the role of gut ontogeny in relation to growth and metabolism.

#### Intestinal Mucosa

The infant’s gut starts off permeable due to its immaturity (63, 64), allowing for increased intestinal absorption of intact proteins compared to the adult (65, 66). This may facilitate the intact absorption of bioactive factors present in breast milk. As touched upon above, from the adult situation it is known that defects in the integrity of the gut barrier can also result in systemic translocation of bacterial/gut-derived material leading to endotoxemia, which contributes to chronic low-grade inflammation and metabolic dysfunction (67–69). In healthy cases a structural barrier of epithelial cells proliferates in early life and restricts the entry of foreign (bacterial) material into the systemic circulation (63, 70). Studies in young infants demonstrate the major beneficial effect of human milk as compared to formula for the development of gut barrier integrity (64, 71). This is most probably related to breast milk factors required for epithelial cell maturation and function, including epidermal growth factor (EGF) (6, 72), IGF-1 (22), transforming growth factor (TGF)-β (73), lactoferrin (74), vaso-active intestinal peptide (VIP) (75), vitamin A (37, 76) and SCFA (36, 37). Analysis of epithelial cells obtained from stool samples at 3 months of age demonstrated differential expression of over 1,000 genes between breastfed and formula-fed infants (77). The importance of the gut barrier in early life for energy metabolism is highlighted in infants with chronic intestinal inflammation. These infants show poor nutrient absorption leading to macro- and micronutrient deficiencies and the use of energy for the inflammatory response, resulting in impaired growth and physical development (78).

A population of innate gut immune cells, innate lymphoid cell type 3 (ILC3), represents a potentially important target for control of energy metabolism by breastfeeding. These cells develop *in utero* under the influence of maternal vitamin A and aryl hydrocarbon receptor (AhR) ligands (79, 80). AhR ligands, originating from the maternal microbiota, are also detected in breast milk and stimulate the development of ILC3 in the intestine of the neonate (80, 81). Mouse models suggest that these cells could be key in the regulation of inflammation and energy metabolism after weaning. Through the secretion of IL-22, ILC3 control the expansion of inflammatory bacteria in the gut (82) and promote a strong epithelial barrier (57), both preventing chronic inflammation. IL-22 also enhances PYY levels, an anorexic gut hormone reducing food intake (57), and is likely involved in the control of lipid uptake. Studies show that IL-22 reduces lipid transport (82) resulting in lower body weight (83) while others describe that IL-22 enhances lipid uptake and export to the adipose tissue, and therefore increase body fat (84). IL-22 secretion by ILC3 is controlled by bacterial activation of Toll-like receptor-MyD88 signaling (46). VIP, secreted from enteric neurons upon food consumption, also appears important for regulation of IL-22-producing ILC3 in the gut. However, divergent effects were observed in two recent studies (83, 85). It may be of interest to investigate the role of VIP in regulating this pathway in early life, as human milk contains ~100pg/mL VIP (86). The studies described here show that various milk components can improve the intestinal barrier and prevent chronic systemic inflammation and thus support adipose tissue homeostasis.

#### Gut Microbiota and Metabolic Health

Perhaps the clearest links between human milk, the gut and metabolic outcomes are related to the gut microbiota. Microbial signals such as microbe-associated molecular patterns (MAMPs) and bacterial metabolites such as SCFA modify local mucosal and systemic immune responses (36), as described above. In the adult, changes in the gut microbiota composition influence energy extraction (87), inflammation (68, 88), metabolic signaling (89) and browning of adipose tissue (90). Because the intestinal microbiota establishes as the adipose tissue and immune system develop, microbiota disruption during the early time window can impair host metabolism, body composition and healthy postnatal growth. Infants with undernutrition show persistent microbiota immaturity (91–93) which correlates with anthropometric growth measurements (93). On the other hand, Caesarean section (94–96), lack of breastfeeding (3) and the use of antibiotics (9–11) are associated with gut dysbiosis and being overweight in childhood. The impact of early life antibiotics on adiposity can remain after ceasing of antibiotic treatment, even though the microbiota recovers, highlighting that alterations in the critical window can have long-term effects (97, 98).

Breastfeeding status is the most significant factor associated with microbiota structure in early life (99–104). Breast milk bacteria contribute to the seeding of the infants gut microbiota, and this is dependent on breastfeeding exclusivity and duration (102). These bacteria most likely originate from the mother as well as other exogenous sources such as the home environment and infant mouth (102). Bacteria transferred through human milk include species such as *Rothia*, *Veillonella* and *Bifidobacterium* that can influence immune homeostasis (102). The microbiota of healthy, exclusively breastfed infants is dominated by *Bifidobacterium*, which is linked to health benefits such as improved gut barrier function (92). A recent cohort showed that the abundance of *Enterococcus* in the infant gut, seemingly due to the lower abundance in maternal breast milk, inversely correlated with infant body weight and fat as well as leptin levels (105). A recent mouse study identified bile acids as potent drivers of intestinal microbial maturation in early life (106), and potentially human milk bile acids may contribute to this effect. Human milk also provides the neonate with large amounts of antimicrobial factors such as lactoferrin, which can further shape the early microbiota (103). Of large interest to date are prebiotic factors such as human milk oligosaccharides (HMOs), which support the growth of beneficial bacteria and can alter colonization patterns in the neonate (103). HMOs are a group of complex sugars that are the third most abundant solid component of human milk, and though non-nutritive to the infant they have many properties besides their role as prebiotics. The influence of HMOs on growth is therefore most probably multi-factorial and beyond its prebiotic activity includes inhibition of pathogen adhesion and host invasion, local and systemic anti-inflammatory effects (107) and modification of the metabolism in the microbiota as well as the host (108). A study amongst Malawian mothers with undernourished infants has demonstrated that milk oligosaccharides are less abundant in breast milk of mothers with stunted infants. This study also showed a causal, microbiota-dependent relationship between sialylated milk oligosaccharides and growth promotion in malnourished animals (108). An elegant mouse study by Cowardin et al., demonstrated that sialylated milk oligosaccharides, but not the most abundant HMO 2’fucosyllactose (2’FL), decreased bone resorption, leading to an increase in bone volume and growth in a similar low resource setting (109). In a healthy population, human milk 2’FL was positively associated with growth in infancy and early childhood, whereas high concentrations of lacto-N-neo-tetraose (LNnT) were associated with reduced body fat and growth (110–112). Overall, HMO diversity was inversely associated with infant fat mass (110) and childhood growth (112). Maternal exercise induced 3’-siallyllactose in human milk, and this HMO improved metabolic health and protected from the harmful effects of high-fat diet feeding in adult mouse offspring (113). These recent studies all point out the importance of HMOs for healthy growth in early life.

## Perspective

Studies addressing the link between human milk composition and infant growth are important to strengthen our understanding of both the short- and long-term health-promoting effects of breast milk. Here we demonstrate that various nutritional, bioactive, growth and immunological factors in human milk may play a role in healthy growth and adipose tissue development in infants. The mechanisms that mediate these positive effects of breastfeeding on growth outcomes remain unclear but the evidence there is today shows this may be related to strengthening the gut barrier and microbiota development, facilitating immune and metabolic homeostasis.

### Future Research

As both the rates of undernutrition and overweight are alarmingly high (114–116), it is important to identify interventions that effectively promote healthy growth of children. Research on the role of human milk on adipose tissue development is scarce. There are large gaps in knowledge on the direct and indirect impact of human milk and breastfeeding on adipogenesis, adipose tissue expansion, beiging of adipose tissue as well as the immune function of the fat depot that deserves to be filled.

The engagement of specific metabolic pathways profoundly affects immune cell differentiation and function. Metabolic programming is, amongst other factors, controlled by the availability of nutrients. Identifying the metabolic requirements and specific nutrients of immune cells in the intestine and adipose tissue in early life, could help to further understand immune homeostasis and healthy growth in infancy. Recently Met-flow (117) has come in the picture as an accessible technique capturing the metabolic state of immune cells that may help answer this question in more detail.

The dynamic composition of human milk, varying significantly within a feed, diurnally and over the course of lactation may play an important role in the healthy development of the infant. This topic needs to be further explored for better understanding of the early diet and healthy growth. Recent evidence point towards the cyclic regulation of ILC3 cells in the intestine, which is linked to the circadian clock as well as food intake (83, 85). As the newborn gut is dominated by innate immune cells (118), the dynamic composition of milk may be of importance for optimal immune development in the intestine and beyond, contributing to metabolic homeostasis.

### Conclusion

The interplay between the gut microbiota, gut mucosa and adipose tissue metabolism is the focus of much research in the adult. This mini-review highlights how this crosstalk might be crucial for a healthy start in life and the key role human milk may play in driving this. Future research will need to replace extrapolation from the adult by evidence gained in the newborn. This will allow the development of (human milk-inspired) child-tailored approaches for metabolic disease prevention.

## Author Contributions

LV and VV wrote, edited, and revised the manuscript together. All authors contributed to the article and approved the submitted version.

## Funding

This work was supported by funding from The University of Western Australia and the Larsson-Rosenquist Foundation.

## Conflict of Interest

The authors declare that the research was conducted in the absence of any commercial or financial relationships that could be construed as a potential conflict of interest.

## References

[1] Victora CG Bahl R Barros AJ França GVA Horton S Krasevec J. Breastfeeding in the 21st century: epidemiology, mechanisms, and lifelong effect. Lancet (2016) 387):475–90. 10.1016/S0140-6736(15)01024-7 26869575

[2] McGuire W Anthony MY. Donor human milk versus formula for preventing necrotising enterocolitis in preterm infants: systematic review. Arch Dis Child Fetal Neonatal Ed (2003) 88(1):F11–4. 10.1136/fn.88.1.f11 PMC1756003 12496220

[3] Forbes JD Azad MB Vehling L Tun HM Konya TB Guttman DS. Association of Exposure to Formula in the Hospital and Subsequent Infant Feeding Practices With Gut Microbiota and Risk of Overweight in the First Year of Life. JAMA Pediatr (2018) 172(7):e181161. 10.1001/jamapediatrics.2018.1161 29868719 PMC6137517

[4] Mazzocchi A Gianni ML Morniroli D Leone L Roggero P Agostoni C. Hormones in Breast Milk and Effect on Infants’ Growth: A Systematic Review. Nutrients (2019) 11(8):1845. 10.3390/nu11081845 PMC6724322 31395844

[5] Lind MV Larnkjaer A Molgaard C Michaelsen KF. Breastfeeding, Breast Milk Composition, and Growth Outcomes. Nestle Nutr Inst Workshop Ser (2018) 89:63–77. 10.1159/000486493 29991033

[6] Ballard O Morrow AL. Human milk composition: nutrients and bioactive factors. Pediatr Clin North Am (2013) 60(1):49–74. 10.1016/j.pcl.2012.10.002 23178060 PMC3586783

[7] Samuel TM Zhou Q Giuffrida F Munblit D Verhasselt V Thakkar SK. Nutritional and Non-nutritional Composition of Human Milk Is Modulated by Maternal, Infant, and Methodological Factors. Front Nutr (2020) 7:576133. 10.3389/fnut.2020.576133 33117843 PMC7557356

[8] Stephensen CB. Burden of infection on growth failure. J Nutr (1999) 129(2S Suppl):534S–8S. 10.1093/jn/129.2.534S 10064326

[9] Ajslev TA Andersen CS Gamborg M Sorensen TI Jess T. Childhood overweight after establishment of the gut microbiota: the role of delivery mode, pre-pregnancy weight and early administration of antibiotics. Int J Obes (2011) 35(4):522–9. 10.1038/ijo.2011.27 21386800

[10] Murphy R Stewart AW Braithwaite I Beasley R Hancox RJ Mitchell EA. Antibiotic treatment during infancy and increased body mass index in boys: an international cross-sectional study. Int J Obes (2014) 38(8):1115–9. 10.1038/ijo.2013.218 24257411

[11] Trasande L Blustein J Liu M Corwin E Cox LM Blaser MJ. Infant antibiotic exposures and early-life body mass. Int J Obes (2013) 37(1):16–23. 10.1038/ijo.2012.132 PMC3798029 22907693

[12] Hassiotou F Geddes DT. Programming of appetite control during breastfeeding as a preventative strategy against the obesity epidemic. J Hum Lact (2014) 30(2):136–42. 10.1177/0890334414526950 24646683

[13] Haschke F Haiden N Thakkar SK. Nutritive and Bioactive Proteins in Breastmilk. Ann Nutr Metab (2016) 69(Suppl 2):17–26. 10.1159/000452820 28103610

[14] Lindquist S Hernell O. Lipid digestion and absorption in early life: an update. Curr Opin Clin Nutr Metab Care (2010) 13(3):314–20. 10.1097/MCO.0b013e328337bbf0 20179589

[15] Forsyth JS Ross PE Bouchier IA. Bile salts in breast milk. Eur J Pediatr (1983) 140(2):126–7. 10.1007/BF00441660 6884389

[16] Selwyn FP Csanaky IL Zhang Y Klaassen CD. Importance of Large Intestine in Regulating Bile Acids and Glucagon-Like Peptide-1 in Germ-Free Mice. Drug Metab Dispos (2015) 43(10):1544–56. 10.1146/annurev.physiol.63.1.141 PMC4576674 26199423

[17] Butler AA Le Roith D. Control of growth by the somatropic axis: growth hormone and the insulin-like growth factors have related and independent roles. Annu Rev Physiol (2001) 63:141–64. 10.1146/annurev.physiol.63.1.141 11181952

[18] Kaplan SA Cohen P. The somatomedin hypothesis 50 years later. J Clin Endocrinol Metab (2007) 92(12):4529–35. 10.1210/jc.2007-0526 17986643

[19] Schwarzer M Makki K Storelli G Machuca-Gayet I Srutkova D Hermanova P. Lactobacillus plantarum strain maintains growth of infant mice during chronic undernutrition. Science (2016) 351(6275):854–7. 10.1126/science.aad8588 26912894

[20] Ontsouka EC Albrecht C Bruckmaier RM. Invited review: Growth-promoting effects of colostrum in calves based on interaction with intestinal cell surface receptors and receptor-like transporters. J Dairy Sci (2016) 99(6):4111–23. 10.3168/jds.2015-9741 26874414

[21] Galante L Pundir S Lagstrom H Rautava S Reynolds CM Milan AM. Growth Factor Concentrations in Human Milk Are Associated With Infant Weight and BMI From Birth to 5 Years. Front Nutr (2020) 7:110. 10.3389/fnut.2020.00110 32850934 PMC7403458

[22] Hoeflich A Meyer Z. Functional analysis of the IGF-system in milk. Best Pract Res Clin Endocrinol Metab (2017) 31(4):409–18. 10.1016/j.beem.2017.10.002 29221569

[23] Poissonnet CM Burdi AR Garn SM. The chronology of adipose tissue appearance and distribution in the human fetus. Early Hum Dev (1984) 10(1-2):1–11. 10.1016/0378-3782(84)90106-3 6499712

[24] Makki K Froguel P Wolowczuk I. Adipose tissue in obesity-related inflammation and insulin resistance: cells, cytokines, and chemokines. ISRN Inflammation (2013) 2013:139239. 10.1155/2013/139239 24455420 PMC3881510

[25] Ikeda K Maretich P Kajimura S. The Common and Distinct Features of Brown and Beige Adipocytes. Trends Endocrinol Metab (2018) 29(3):191–200. 10.1016/j.tem.2018.01.001 29366777 PMC5826798

[26] Symonds ME Bloor I Ojha S Budge H. The Placenta, Maternal Diet and Adipose Tissue Development in the Newborn. Ann Nutr Metab (2017) 70(3):232–5. 10.1159/000464301 28301844

[27] Ghaben AL Scherer PE. Adipogenesis and metabolic health. Nat Rev Mol Cell Biol (2019) 20(4):242–58. 10.1038/s41580-018-0093-z 30610207

[28] Budge H Sebert S Sharkey D Symonds ME. Session on ‘Obesity’. Adipose tissue development, nutrition in early life and its impact on later obesity. Proc Nutr Soc (2009) 68(3):321–6. 10.1017/S0029665109001402 19490741

[29] Tang W Zeve D Suh JM Bosnakovski D Kyba M Hammer RE. White fat progenitor cells reside in the adipose vasculature. Science (2008) 322(5901):583–6. 10.1126/science.1156232 PMC2597101 18801968

[30] Palhinha L Liechocki S Hottz ED Pereira J de Almeida CJ Moraes-Vieira PMM. Leptin Induces Proadipogenic and Proinflammatory Signaling in Adipocytes. Front Endocrinol (2019) 10:841. 10.3389/fendo.2019.00841 PMC6923660 31920961

[31] Fu Y Luo N Klein RL Garvey WT. Adiponectin promotes adipocyte differentiation, insulin sensitivity, and lipid accumulation. J Lipid Res (2005) 46(7):1369–79. 10.1194/jlr.M400373-JLR200 15834118

[32] Moreno-Navarrete JM Ortega F Moreno M Serrano M Ricart W Fernandez-Real JM. Lactoferrin gene knockdown leads to similar effects to iron chelation in human adipocytes. J Cell Mol Med (2014) 18(3):391–5. 10.1111/jcmm.12234 PMC3955146 24571258

[33] Hong YH Nishimura Y Hishikawa D Tsuzuki H Miyahara H Gotoh C. Acetate and propionate short chain fatty acids stimulate adipogenesis *via* GPCR43. Endocrinology (2005) 146(12):5092–9. 10.1210/en.2005-0545 16123168

[34] Li G Yao W Jiang H. Short-chain fatty acids enhance adipocyte differentiation in the stromal vascular fraction of porcine adipose tissue. J Nutr (2014) 144(12):1887–95. 10.3945/jn.114.198531 25320182

[35] Stinson LF Gay MCL Koleva PT Eggesbo M Johnson CC Wegienka G. Human Milk From Atopic Mothers Has Lower Levels of Short Chain Fatty Acids. Front Immunol (2020) 11:1427. 10.3389/fimmu.2020.01427 32903327 PMC7396598

[36] Rooks MG Garrett WS. Gut microbiota, metabolites and host immunity. Nat Rev Immunol (2016) 16(6):341–52. 10.1038/nri.2016.42 PMC5541232 27231050

[37] van den Elsen LWJ Rekima A Verhasselt V. Early-Life Nutrition and Gut Immune Development. Nestle Nutr Inst Workshop Ser (2019) 90:137–49. 10.1159/000490301 30865982

[38] Lacasa D Taleb S Keophiphath M Miranville A Clement K. Macrophage-secreted factors impair human adipogenesis: involvement of proinflammatory state in preadipocytes. Endocrinology (2007) 148(2):868–77. 10.1210/en.2006-0687 17082259

[39] Jiang N Li Y Shu T Wang J. Cytokines and inflammation in adipogenesis: an updated review. Front Med (2019) 13(3):314–29. 10.1007/s11684-018-0625-0 30066061

[40] Luo L Liu M. Adipose tissue in control of metabolism. J Endocrinol (2016) 231(3):R77–99. 10.1530/JOE-16-0211 PMC7928204 27935822

[41] Meln I Wolff G Gajek T Koddebusch J Lerch S Harbrecht L. Dietary calories and lipids synergistically shape adipose tissue cellularity during postnatal growth. Mol Metab (2019) 24:139–48. 10.1016/j.molmet.2019.03.012 PMC6531874 31003943

[42] Tulp O Gambert S Horton ES. Adipose tissue development, growth, and food consumption in protein-malnourished rats. J Lipid Res (1979) 20(1):47–54. 10.1016/S0022-2275(20)40650-9 108348

[43] Kozak LP Koza RA Anunciado-Koza R Mendoza T Newman S. Inherent plasticity of brown adipogenesis in white fat of mice allows for recovery from effects of post-natal malnutrition. PloS One (2012) 7(2):e30392. 10.1371/journal.pone.0030392 22383960 PMC3286483

[44] Claycombe KJ Vomhof-DeKrey EE Garcia R Johnson WT Uthus E Roemmich JN. Decreased beige adipocyte number and mitochondrial respiration coincide with increased histone methyl transferase (G9a) and reduced FGF21 gene expression in Sprague-Dawley rats fed prenatal low protein and postnatal high-fat diets. J Nutr Biochem (2016) 31:113–21. 10.1016/j.jnutbio.2016.01.008 27133430

[45] Wang J Ge J Cao H Zhang X Guo Y Li X. Leptin Promotes White Adipocyte Browning by Inhibiting the Hh Signaling Pathway. Cells (2019) 8(4):372. 10.3390/cells8040372 PMC6523697 31022919

[46] Wei Q Lee JH Wang H Bongmba OYN Wu CS Pradhan G. Adiponectin is required for maintaining normal body temperature in a cold environment. BMC Physiol (2017) 17(1):8. 10.1186/s12899-017-0034-7 29058611 PMC5651620

[47] Nakamura K Kishida T Ejima A Tateyama R Morishita S Ono T. Bovine lactoferrin promotes energy expenditure *via* the cAMP-PKA signaling pathway in human reprogrammed brown adipocytes. Biometals (2018) 31(3):415–24. 10.1007/s10534-018-0103-9 29744695

[48] Yu H Dilbaz S Cossmann J Hoang AC Diedrich V Herwig A. Breast milk alkylglycerols sustain beige adipocytes through adipose tissue macrophages. J Clin Invest (2019) 129(6):2485–99. 10.1172/JCI125646 PMC6546455 31081799

[49] Rockstroh D Landgraf K Wagner IV Gesing J Tauscher R Lakowa N. Direct evidence of brown adipocytes in different fat depots in children. PloS One (2015) 10(2):e0117841. 10.1371/journal.pone.0117841 25706927 PMC4338084

[50] Prentice PM Schoemaker MH Vervoort J Hettinga K Lambers TT van Tol EAF. Human Milk Short-Chain Fatty Acid Composition is Associated with Adiposity Outcomes in Infants. J Nutr (2019) 149(5):716–22. 10.1093/jn/nxy320 31050748

[51] Gao Z Yin J Zhang J Ward RE Martin RJ Lefevre M. Butyrate improves insulin sensitivity and increases energy expenditure in mice. Diabetes (2009) 58(7):1509–17. 10.2337/db08-1637 PMC2699871 19366864

[52] Li Z Yi CX Katiraei S Kooijman S Zhou E Chung CK. Butyrate reduces appetite and activates brown adipose tissue *via* the gut-brain neural circuit. Gut (2018) 67(7):1269–79. 10.1136/gutjnl-2017-314050 29101261

[53] Campbell C Rudensky A. Roles of Regulatory T Cells in Tissue Pathophysiology and Metabolism. Cell Metab (2020) 31(1):18–25. 10.1016/j.cmet.2019.09.010 31607562 PMC7657366

[54] Feuerer M Herrero L Cipolletta D Naaz A Wong J Nayer A. Lean, but not obese, fat is enriched for a unique population of regulatory T cells that affect metabolic parameters. Nat Med (2009) 15(8):930–9. 10.1038/nm.2002 PMC3115752 19633656

[55] Everaere L Ait Yahia S Boute M Audousset C Chenivesse C Tsicopoulos A. Innate lymphoid cells at the interface between obesity and asthma. Immunology (2018) 153(1):21–30. 10.1111/imm.12832 28880992 PMC5721241

[56] Lee MW Odegaard JI Mukundan L Qiu Y Molofsky AB Nussbaum JC. Activated type 2 innate lymphoid cells regulate beige fat biogenesis. Cell (2015) 160(1-2):74–87. 10.1016/j.cell.2014.12.011 25543153 PMC4297518

[57] Wang X Ota N Manzanillo P Kates L Zavala-Solorio J Eidenschenk C. Interleukin-22 alleviates metabolic disorders and restores mucosal immunity in diabetes. Nature (2014) 514(7521):237–41. 10.1038/nature13564 25119041

[58] van den Elsen L Garssen J Willemsen L. Long chain N-3 polyunsaturated fatty acids in the prevention of allergic and cardiovascular disease. Curr Pharm Des (2012) 18(16):2375–92. 10.2174/138161212800165960 22390701

[59] Oh DY Talukdar S Bae EJ Imamura T Morinaga H Fan W. GPR120 is an omega-3 fatty acid receptor mediating potent anti-inflammatory and insulin-sensitizing effects. Cell (2010) 142(5):687–98. 10.1016/j.cell.2010.07.041 PMC2956412 20813258

[60] Roelofsen H Priebe MG Vonk RJ. The interaction of short-chain fatty acids with adipose tissue: relevance for prevention of type 2 diabetes. Benef Microbes (2010) 1(4):433–7. 10.3920/BM2010.0028 21831781

[61] Vinolo MA Rodrigues HG Festuccia WT Crisma AR Alves VS Martins AR. Tributyrin attenuates obesity-associated inflammation and insulin resistance in high-fat-fed mice. Am J Physiol Endocrinol Metab (2012) 303(2):E272–82. 10.1152/ajpendo.00053.2012 22621868

[62] La Cava A Matarese G. The weight of leptin in immunity. Nat Rev Immunol (2004) 4(5):371–9. 10.1038/nri1350 15122202

[63] de Santa Barbara P van den Brink GR Roberts DJ. Development and differentiation of the intestinal epithelium. Cell Mol Life Sci (2003) 60(7):1322–32. 10.1007/s00018-003-2289-3 PMC2435618 12943221

[64] Catassi C Bonucci A Coppa GV Carlucci A Giorgi PL. Intestinal permeability changes during the first month: effect of natural versus artificial feeding. J Pediatr Gastroenterol Nutr (1995) 21(4):383–6. 10.1097/00005176-199511000-00003 8583288

[65] Heyman M Crain-Denoyelle AM Corthier G Morgat JL Desjeux JF. Postnatal development of protein absorption in conventional and germ-free mice. Am J Physiol (1986) 251(3 Pt 1):G326–31. 10.1152/ajpgi.1986.251.3.G326 3752248

[66] Castell JV Friedrich G Kuhn CS Poppe GE. Intestinal absorption of undegraded proteins in men: presence of bromelain in plasma after oral intake. Am J Physiol (1997) 273(1 Pt 1):G139–46. 10.1152/ajpgi.1997.273.1.G139 9252520

[67] Cani PD Amar J Iglesias MA Poggi M Knauf C Bastelica D. Metabolic endotoxemia initiates obesity and insulin resistance. Diabetes (2007) 56(7):1761–72. 10.2337/db06-1491 17456850

[68] Cani PD Bibiloni R Knauf C Waget A Neyrinck AM Delzenne NM. Changes in gut microbiota control metabolic endotoxemia-induced inflammation in high-fat diet-induced obesity and diabetes in mice. Diabetes (2008) 57(6):1470–81. 10.2337/db07-1403 18305141

[69] Burcelin R Serino M Chabo C Garidou L Pomie C Courtney M. Metagenome and metabolism: the tissue microbiota hypothesis. Diabetes Obes Metab (2013) 15(Suppl 3):61–70. 10.1111/dom.12157 24003922

[70] Henning SJ. Postnatal development: coordination of feeding, digestion, and metabolism. Am J Physiol (1981) 241(3):G199–214. 10.1152/ajpgi.1981.241.3.G199 7025659

[71] Taylor SN Basile LA Ebeling M Wagner CL. Intestinal permeability in preterm infants by feeding type: mother’s milk versus formula. Breastfeed Med (2009) 4(1):11–5. 10.1089/bfm.2008.0114 PMC2932544 19196035

[72] Tang X Liu H Yang S Li Z Zhong J Fang R. Epidermal Growth Factor and Intestinal Barrier Function. Mediators Inflammation (2016) 2016:1927348. 10.1155/2016/1927348 PMC4976184 27524860

[73] Turfkruyer M Verhasselt V. Breast milk and its impact on maturation of the neonatal immune system. Curr Opin Infect Dis (2015) 28(3):199–206. 10.1097/QCO.0000000000000165 25887614

[74] Liao Y Jiang R Lonnerdal B. Biochemical and molecular impacts of lactoferrin on small intestinal growth and development during early life. Biochem Cell Biol (2012) 90(3):476–84. 10.1139/o11-075 22332905

[75] Iwasaki M Akiba Y Kaunitz JD. Recent advances in vasoactive intestinal peptide physiology and pathophysiology: focus on the gastrointestinal system. F1000Res (2019) 8:F1000 Faculty Rev-1629. 10.12688/f1000research.18039.1 PMC6743256 31559013

[76] Turfkruyer M Rekima A Macchiaverni P Le Bourhis L Muncan V van den Brink GR. Oral tolerance is inefficient in neonatal mice due to a physiological vitamin A deficiency. Mucosal Immunol (2016) 9(2):479–91. 10.1038/mi.2015.114 26530133

[77] Chapkin RS Zhao C Ivanov I Davidson LA Goldsby JS Lupton JR. Noninvasive stool-based detection of infant gastrointestinal development using gene expression profiles from exfoliated epithelial cells. Am J Physiol Gastrintest Liver Physiol (2010) 298(5):G582–9. 10.1152/ajpgi.00004.2010 PMC2867429 20203060

[78] Ali A Iqbal NT Sadiq K. Environmental enteropathy. Curr Opin Gastroenterol (2016) 32(1):12–7. 10.1097/MOG.0000000000000226 26574871

[79] van de Pavert SA Ferreira M Domingues RG Ribeiro H Molenaar R Moreira-Santos L. Maternal retinoids control type 3 innate lymphoid cells and set the offspring immunity. Nature (2014) 508(7494):123–7. 10.1038/nature13158 PMC4932833 24670648

[80] Gomez de Aguero M Ganal-Vonarburg SC Fuhrer T Rupp S Uchimura Y Li H. The maternal microbiota drives early postnatal innate immune development. Science (2016) 351(6279):1296–302. 10.1126/science.aad2571 26989247

[81] Torow N Marsland BJ Hornef MW Gollwitzer ES. Neonatal mucosal immunology. Mucosal Immunol (2017) 10(1):5–17. 10.1038/mi.2016.81 27649929

[82] Mao K Baptista AP Tamoutounour S Zhuang L Bouladoux N Martins AJ. Innate and adaptive lymphocytes sequentially shape the gut microbiota and lipid metabolism. Nature (2018) 554(7691):255–9. 10.1038/nature25437 29364878

[83] Talbot J Hahn P Kroehling L Nguyen H Li D Littman DR. Feeding-dependent VIP neuron-ILC3 circuit regulates the intestinal barrier. Nature (2020) 579(7800):575–80. 10.1038/s41586-020-2039-9 PMC7135938 32050257

[84] Wang Y Kuang Z Yu X Ruhn KA Kubo M Hooper LV. The intestinal microbiota regulates body composition through NFIL3 and the circadian clock. Science (2017) 357(6354):912–6. 10.1126/science.aan0677 PMC5702268 28860383

[85] Seillet C Luong K Tellier J Jacquelot N Shen RD Hickey P. The neuropeptide VIP confers anticipatory mucosal immunity by regulating ILC3 activity. Nat Immunol (2020) 21(2):168–77. 10.1038/s41590-019-0567-y 31873294

[86] Werner H Koch Y Fridkin M Fahrenkrug J Gozes I. High levels of vasoactive intestinal peptide in human milk. Biochem Biophys Res Commun (1985) 133(1):228–32. 10.1016/0006-291x(85)91865-0 4074363

[87] Turnbaugh PJ Ley RE Mahowald MA Magrini V Mardis ER Gordon JI. An obesity-associated gut microbiome with increased capacity for energy harvest. Nature (2006) 444(7122):1027–31. 10.1038/nature05414 17183312

[88] Vijay-Kumar M Aitken JD Carvalho FA Cullender TC Mwangi S Srinivasan S. Metabolic syndrome and altered gut microbiota in mice lacking Toll-like receptor 5. Science (2010) 328(5975):228–31. 10.1126/science.1179721 PMC4714868 20203013

[89] Samuel BS Shaito A Motoike T Rey FE Backhed F Manchester JK. Effects of the gut microbiota on host adiposity are modulated by the short-chain fatty-acid binding G protein-coupled receptor, Gpr41. Proc Natl Acad Sci U.S.A. (2008) 105(43):16767–72. 10.1073/pnas.0808567105 PMC2569967 18931303

[90] Suarez-Zamorano N Fabbiano S Chevalier C Stojanovic O Colin DJ Stevanovic A. Microbiota depletion promotes browning of white adipose tissue and reduces obesity. Nat Med (2015) 21(12):1497–501. 10.1038/nm.3994 PMC4675088 26569380

[91] Kau AL Ahern PP Griffin NW Goodman AL Gordon JI. Human nutrition, the gut microbiome and the immune system. Nature (2011) 474(7351):327–36. 10.1038/nature10213 PMC3298082 21677749

[92] Subramanian S Blanton LV Frese SA Charbonneau M Mills DA Gordon JI. Cultivating healthy growth and nutrition through the gut microbiota. Cell (2015) 161(1):36–48. 10.1016/j.cell.2015.03.013 25815983 PMC4440586

[93] Subramanian S Huq S Yatsunenko T Haque R Mahfuz M Alam MA. Persistent gut microbiota immaturity in malnourished Bangladeshi children. Nature (2014) 510(7505):417–21. 10.1038/nature13421 PMC4189846 24896187

[94] Dominguez-Bello MG Costello EK Contreras M Magris M Hidalgo G Fierer N. Delivery mode shapes the acquisition and structure of the initial microbiota across multiple body habitats in newborns. Proc Natl Acad Sci U.S.A. (2010) 107(26):11971–5. 10.1073/pnas.1002601107 PMC2900693 20566857

[95] Blustein J Attina T Liu M Ryan AM Cox LM Blaser MJ. Association of caesarean delivery with child adiposity from age 6 weeks to 15 years. Int J Obes (2013) 37(7):900–6. 10.1038/ijo.2013.49 PMC5007946 23670220

[96] Huh SY Rifas-Shiman SL Zera CA Edwards JW Oken E Weiss ST. Delivery by caesarean section and risk of obesity in preschool age children: a prospective cohort study. Arch Dis Childh (2012) 97(7):610–6. 10.1136/archdischild-2011-301141 PMC3784307 22623615

[97] Cho I Yamanishi S Cox L Methe BA Zavadil J Li K. Antibiotics in early life alter the murine colonic microbiome and adiposity. Nature (2012) 488(7413):621–6. 10.1038/nature11400 PMC3553221 22914093

[98] Cox LM Yamanishi S Sohn J Alekseyenko AV Leung JM Cho I. Altering the intestinal microbiota during a critical developmental window has lasting metabolic consequences. Cell (2014) 158(4):705–21. 10.1016/j.cell.2014.05.052 PMC4134513 25126780

[99] Backhed F Roswall J Peng Y Feng Q Jia H Kovatcheva-Datchary P. Dynamics and Stabilization of the Human Gut Microbiome during the First Year of Life. Cell Host Microbe (2015) 17(5):690–703. 10.1016/j.chom.2015.04.004 25974306

[100] Stewart CJ Ajami NJ O’Brien JL Hutchinson DS Smith DP Wong MC. Temporal development of the gut microbiome in early childhood from the TEDDY study. Nature (2018) 562(7728):583–8. 10.1038/s41586-018-0617-x PMC6415775 30356187

[101] Pannaraj PS Li F Cerini C Bender JM Yang S Rollie A. Association Between Breast Milk Bacterial Communities and Establishment and Development of the Infant Gut Microbiome. JAMA Pediatr (2017) 171(7):647–54. 10.1001/jamapediatrics.2017.0378 PMC5710346 28492938

[102] Fehr K Moossavi S Sbihi H Boutin RCT Bode L Robertson B. Breastmilk Feeding Practices Are Associated with the Co-Occurrence of Bacteria in Mothers’ Milk and the Infant Gut: the CHILD Cohort Study. Cell Host Microbe (2020) 28(2):285–97 e4. 10.1016/j.chom.2020.06.009 32652062

[103] van den Elsen LWJ Garssen J Burcelin R Verhasselt V. Shaping the Gut Microbiota by Breastfeeding: The Gateway to Allergy Prevention? Front Pediatr (2019) 7:47. 10.3389/fped.2019.00047 30873394 PMC6400986

[104] Galazzo G van Best N Bervoets L Dapaah IO Savelkoul PH Hornef MW. Development of the Microbiota and Associations With Birth Mode, Diet, and Atopic Disorders in a Longitudinal Analysis of Stool Samples, Collected From Infancy Through Early Childhood. Gastroenterology (2020) 158(6):1584–96. 10.1053/j.gastro.2020.01.024 31958431

[105] Laursen MF Larsson MW Lind MV Larnkjaer A Molgaard C Michaelsen KF. Intestinal Enterococcus abundance correlates inversely with excessive weight gain and increased plasma leptin in breastfed infants. FEMS Microbiol Ecol (2020) 96(5):fiaa066. 10.1093/femsec/fiaa066 32275305 PMC7183236

[106] van Best N Rolle-Kampczyk U Schaap FG Basic M Olde Damink SWM Bleich A. Bile acids drive the newborn’s gut microbiota maturation. Nat Commun (2020) 11(1):3692. 10.1038/s41467-020-17183-8 32703946 PMC7378201

[107] He Y Liu S Leone S Newburg DS. Human colostrum oligosaccharides modulate major immunologic pathways of immature human intestine. Mucosal Immunol (2014) 7(6):1326–39. 10.1038/mi.2014.20 PMC4183735 24691111

[108] Charbonneau MR O’Donnell D Blanton LV Totten SM Davis JC Barratt MJ. Sialylated Milk Oligosaccharides Promote Microbiota-Dependent Growth in Models of Infant Undernutrition. Cell (2016) 164(5):859–71. 10.1016/j.cell.2016.01.024 PMC4793393 26898329

[109] Cowardin CA Ahern PP Kung VL Hibberd MC Cheng J Guruge JL. Mechanisms by which sialylated milk oligosaccharides impact bone biology in a gnotobiotic mouse model of infant undernutrition. Proc Natl Acad Sci U.S.A. (2019) 116(24):11988–96. 10.1073/pnas.1821770116 PMC6575181 31138692

[110] Alderete TL Autran C Brekke BE Knight R Bode L Goran MI. Associations between human milk oligosaccharides and infant body composition in the first 6 mo of life. Am J Clin Nutr (2015) 102(6):1381–8. 10.3945/ajcn.115.115451 PMC6546222 26511224

[111] Larsson MW Lind MV Laursen RP Yonemitsu C Larnkjaer A Molgaard C. Human Milk Oligosaccharide Composition Is Associated With Excessive Weight Gain During Exclusive Breastfeeding-An Explorative Study. Front Pediatr (2019) 7:297. 10.3389/fped.2019.00297 31380329 PMC6657391

[112] Lagstrom H Rautava S Ollila H Kaljonen A Turta O Makela J. Associations between human milk oligosaccharides and growth in infancy and early childhood. Am J Clin Nutr (2020) 111(4):769–78. 10.1093/ajcn/nqaa010 PMC7138667 32068776

[113] Harris JE Pinckard KM Wright KR Baer LA Arts PJ Abay E. Exercise-induced 3’-sialyllactose in breast milk is a critical mediator to improve metabolic health and cardiac function in mouse offspring. Nat Metab (2020) 2(8):678–87. 10.1016/S0140-6736(19)32497-3 PMC7438265 32694823

[114] Popkin BM Corvalan C Grummer-Strawn LM. Dynamics of the double burden of malnutrition and the changing nutrition reality. Lancet (2020) 395(10217):65–74. 10.1016/S0140-6736(19)32497-3 31852602 PMC7179702

[115] Wells JC Sawaya AL Wibaek R Mwangome M Poullas MS Yajnik CS. The double burden of malnutrition: aetiological pathways and consequences for health. Lancet (2020) 395(10217):75–88. 10.1016/S0140-6736(19)32472-9 31852605 PMC7613491

[116] Unicef. Unicef Data (2020). Available at: https://data.unicef.org/topic/nutrition/malnutrition/ (Accessed December 22, 2020).

[117] Ahl PJ Hopkins RA Xiang WW Au B Kaliaperumal N Fairhurst AM. Met-Flow, a strategy for single-cell metabolic analysis highlights dynamic changes in immune subpopulations. Commun Biol (2020) 3(1):305. 10.1038/s42003-020-1027-9 32533056 PMC7292829

[118] Diefenbach A Gnafakis S Shomrat O. Innate Lymphoid Cell-Epithelial Cell Modules Sustain Intestinal Homeostasis. Immunity (2020) 52(3):452–63. 10.1016/j.immuni.2020.02.016 32187516

